# Impact of large-scale distribution and subsequent use of free nicotine patches on primary care physician interaction

**DOI:** 10.1186/s12889-017-4548-5

**Published:** 2017-07-11

**Authors:** Vladyslav Kushnir, Beth A. Sproule, John A. Cunningham

**Affiliations:** 1 Centre for Addiction and Mental Health, 33 Russell St, Toronto, Ontario M5S 2S1 Canada; 2 Leslie Dan Faculty of Pharmacy, University of Toronto, Toronto, M5S 3M2 Canada; 3 Department of Psychiatry, University of Toronto, Toronto, M5T 1R8 Canada; 4 Research School of Population Health, the Australian National University, Canberra, 2601 Australia

**Keywords:** Smoking cessation, Nicotine replacement therapy, Primary care physicians, Tobacco, Health professionals

## Abstract

**Background:**

Large-scale distribution efforts of free nicotine replacement therapy (NRT) have been documented to be cost-effective interventions for increasing smoking quit rates. However, despite nearly a dozen studies evaluating their effectiveness, none have examined whether free NRT provision promotes further primary care help-seeking and the impact that it may have on cessation efforts.

**Methods:**

In the context of a randomized controlled trial, a secondary analysis was conducted on 1000 adult regular smokers randomized to be mailed a 5-week supply of nicotine patches or to a no intervention control group. Recipients and users of free nicotine patches at an 8 week follow-up were successfully case matched to controls based on age, gender, baseline level of nicotine dependence and intent to quit (*n* = 201 per group). Differences in physician interaction between the two groups were evaluated at both 8 week and 6 month follow-ups. The impact of physician interaction on self-reported smoking abstinence at each follow-up was also examined.

**Results:**

Although no differences in physician interaction were noted between groups at the 8 week follow-up, at the 6 month follow-up, nicotine patch users reported greater frequency of discussing smoking with their physician (43.9%), as compared to the control group (30.3%) (*p* = 0.011). Across both groups, over 90% of those that discussed smoking with a physician were encouraged to quit and approximately 70% were provided with additional support. Separate ANOVAs revealed no significant impact of physician interaction on cessation (*p* > 0.05), regardless of group or follow-up period, however, at the 6 month follow-up, nicotine patch users who discussed cessation with a physician had made serious quit attempts at significantly greater rates (72.6%), compared to controls (49.1%) (*p* = 0.007).

**Conclusions:**

Irrespective of group, the majority of smokers in the present study did not discuss cessation with their physician. Recipients and users of nicotine patches however, were more likely to discuss smoking with their physician, suggesting that the provision of free NRT particularly to those who are likely to use it may facilitate opportunities for benefits beyond the direct pharmacological effects of the medication.

**Trial registration:**

clinicaltrials.gov, NCT01429129. Registered: 2 September 2011.

## Background

Physicians are thought to play a critical role in the provision of smoking cessation assistance to patients [1]. As more than 70% of smokers visit a physician annually [2], medical practitioners are seen to have optimal opportunity to promote smoking cessation to their patients. Primary care practitioners in particular are visited on average more than 4 times per year by patients that smoke, and have thus been directed by clinical practice guidelines to identify and offer support to smokers at every visit. The Clinical Practice Guidelines for Treating Tobacco Use and Dependence, originally developed in 1996 by the United States Department of Human Health Services, stress that primary care physicians should treat tobacco dependence as a chronic disease and follow the 5A’s model of: asking about smoking, assessing readiness to quit, advising smokers to stop, assisting patients with treatment, and arranging follow-up. These forms of intervention have been shown to be quite effective in motivating and driving smokers to quit, where even brief advice increases the odds of cessation [3, 4]. Combined behavioral counseling and pharmacotherapy however, has been shown to be the most effective at treating tobacco dependence, producing the highest odds of quitting and progressively higher quitting estimates with increased number of counseling sessions [5].

Despite evidence showing that smoking cessation intervention in primary care is effective at helping smokers quit and offers a cost-effective option of reaching most smokers [6], studies have shown that physicians have not been active in providing such assistance. Population-based surveys of smokers have revealed that between 44 and 71% were ever advised to quit, and even fewer received any form of intervention [7–9]. One telephone-based population survey had further documented that discussion about smoking was largely dependent on whether smokers were women, in the preparation stage of change, in fair or poor health, and smoked for a greater number of years [9]. With follow-up care also arranged in less than 10% of all visits [8, 9], these practice gaps confirm findings from physician surveys that report physician behavior is below recommended guidelines [10–12].

To address such gaps, researchers have called for specific strategies aimed at enhancing the integration of clinical smoking cessation interventions into primary care settings. Some of these strategies include advanced training initiatives for physicians, enhanced use of established interventions, establishing performance feedback for practitioners, as well as the provision and combined use of low-cost or cost-free pharmacotherapy for patients [10, 13]. While several trials have evaluated the effectiveness of multi-component interventions in primary settings, to date, only one investigated the provision of cost-free medication. The trial revealed that the provision of cost-free nicotine replacement therapy (NRT) or bupropion in combination with general practitioner training strongly increases the odds of cessation (odds ratio of 4.77) and is markedly cost-effective in reducing smoking-related morbidity [14, 15]. As these findings suggest that smoking cessation support and provision of free pharmacotherapy in primary practice is an effective strategy of reducing smoking prevalence in the general population, cost-free medication provision appears to be an important component of achieving higher abstinence rates.

Over the past decade, many giveaway programs outside of clinical settings have provided NRT to large samples of smokers as part of a telephone quitline or large-scale distribution program. Studies evaluating these programs have generally revealed that compared to non-treatment cohorts, smokers receiving NRT were able to achieve higher quit rates, and that offering free NRT is an effective intervention in encouraging program participation, improves treatment satisfaction, and is cost-effective [16–20]. Direct causal evidence on the efficacy of large-scale distribution of free NRT has also been recently established, documenting that despite concerns over the ‘real-world’ effectiveness of NRT [21, 22], the mailed distribution of free nicotine patches in absence of behavioral support more than doubled the odds of cessation at a 6-month follow-up [23]. Nonetheless, no previous studies had evaluated whether the provision of free NRT had promoted further help-seeking and what impact that interaction may have had. As mass distribution of NRT is being considered in many jurisdictions across the United States and Canada, it is important to identify whether this form of intervention drives smokers to seek out additional front-line support.

In the context of a randomized controlled trial evaluating the efficacy of mass distribution of free NRT to smokers, the aim of this study was to examine whether and to what extent the provision of free NRT impacts smokers’ interaction with primary care physicians. In particular, the research attempted to answer the question: does the provision and subsequent use of free nicotine patches to smokers interested in quitting promote interaction with their primary care physicians, and whether that interaction has a role in quitting smoking?

## Methods

### Study design and participants

A detailed research protocol of the overall trial design and primary outcomes are published elsewhere [23, 24]. Briefly, the trial employed a single blinded, panel survey design with random assignment to an experimental and a control group. Random digit dialing of Canadian telephone numbers and an initial interview was used to identify households with adult (age 18 or over) smokers who smoke 10 or more cigarettes per day. One individual from each household who was willing to take part in a smoking study that involved three interviews (baseline, 8-week and 6-month follow-ups) was randomly selected (according to most recent birthday). Of 43,785 households contacted, 2737 contained at least one adult daily smoker, and 2093 consenting individuals were interviewed (response rate of 76.5%) in English or French.

As part of the baseline survey, eligible participants (*n* = 1000) were identified and randomized into experimental and control groups to receive versus not receive free nicotine patches. Eligibility was determined by a series of questions regarding hypothetical interest in nicotine patches to quit smoking (including willingness to have nicotine patches sent to their home) and having no contraindications for using NRT. A randomized half of eligible participants were assigned to the experimental group and asked for their permission to have nicotine patches sent to their home. These participants were sent a package of 5 weeks of nicotine patches (tapered regimen of 3 weeks 21 mg patches, 1 week 14 mg patches, and 1 week 7 mg patches) to their homes via expedited postal mail to help them quit smoking. The package also contained a cover letter instructing them on the use of the patches, a list of answers to frequently asked questions, as well advice to talk to their physician or pharmacist if they had further questions. Participants in the control group were not offered nicotine patches or any other form of support, and were not aware that nicotine patches were offered to others.

### Baseline and follow-up measures

All baseline and follow-up surveys were conducted by trained interviewers from the University of Waterloo Survey Research Centre. In addition to assessing eligibility for the randomized controlled trial, the baseline survey assessed participants’ level of nicotine dependence using the Fagerström Test for Nicotine Dependence (FTND) [25], intent to quit using the Transtheoretical Model’s stages of change (precontemplation, contemplation, and preparation stages) [26, 27], number and duration of past quit attempts, past use of NRT, motivation to quit smoking, a series of demographic characteristics, and the presence of a family practitioner. Those expressing intent to quit in the next 30 days and 6 months (preparation and contemplation stages, respectively) were further asked of their comfort in discussing smoking cessation with their primary care practitioner and whether they perceived their physician was aware of their interest in quitting.

Follow-up surveys conducted at both 8 weeks and 6 months post-baseline assessed smoking status, where abstinence at each of the follow-up periods was measured as positive endorsement of ‘not smoking even a puff’ for at least 7 days or 30 days, respectively. At 8 weeks, participants in the experimental group were asked if and how much of the nicotine patches sent to them were used (assessed using response options of “none”, “some” and “all”), whether they had informed their physician of NRT use, their reasons for and against informing their physician, as well as having received additional support from their physician. Participants in the no-intervention control group were asked whether they had purchased and used any nicotine patches over-the-counter (OTC), had discussed smoking cessation with their physician post-baseline and the support, if any, they had received. At 6 months, all participants regardless of their randomly allocated group were asked if they had talked to their physician about smoking since they were last interviewed, whether they were encouraged by their practitioner to quit, and what forms of smoking cessation intervention were provided to them to help them quit. Discussion of smoking cessation with a primary care practitioner at 6 months post-baseline was the primary outcome measure.

### Analyses

To investigate the impact of free NRT provision and use on physician interaction and smoking cessation, participants in the experimental group who endorsed using at least some of the provided nicotine patches by the 8 week follow-up (*n* = 246) were case matched to participants in the control group based on age, gender, severity of nicotine dependence, stages of change (precontemplation, contemplation, and preparation stages) and the completion of the 8 week follow-up survey. Both age and severity of nicotine dependence as determined by FTND were recoded into categorical values to facilitate optimal case matching. The age variable was recoded into five categories of 18–24, 25–34, 35–44, 45–54, and 55+, while FTND scores of 1 or 2 corresponded to low dependence, scores of 3 or 5 – low to moderate dependence, scores of 5 to 7 – moderate dependence, and scores of 8 to 10 – high dependence. A total of 201 nicotine patch users were case matched to 201 no-intervention control participants.

Group differences in baseline demographic and smoking characteristics were evaluated using one-way analysis of variance (ANOVA) analyses for continuous variables and Pearson’s chi-square tests of independence for categorical variables. In line with recommendations for outcomes analysis of matched case-control data [28, 29], McNemar’s tests were used to evaluate differences in physician interaction at each follow-up, defined as discussing smoking with a physician. These analyses restricted the data to case-controlled pairs and were thus conducted only among those who were followed up and had a family physician at the respective follow-up (8 weeks: 177 matched pairs; 6 months: 155 matched pairs). Among specifically nicotine patch users in the experimental group, within group analyses further used chi square analyses to examine the impact of amount of nicotine patches used on physician interaction. To investigate the impact of physician interaction on cessation outcomes at 8 weeks and 6 months, separate univariate ANOVAs were conducted with group (experimental group nicotine patch users vs. control) as the between subjects factor, physician interaction at each follow-up as the within subjects factor, and making a serious quit attempt (defined as stopping smoking for 1 day or longer) and self-reported smoking abstinence (7-day point prevalence abstinence at 8 weeks and 30-day point-prevalence abstinence at 6 months) as the outcome measures. An intent-to-treat approach was employed for self-reported smoking abstinence, such that all participants lost to follow-up were assumed to be active smoking. All statistical analyses were conducted using IBM SPSS Statistics, version 24.0.

## Results

### Demographic characteristics

Users of freely provided nicotine patches did not differ from case matched controls on any demographic or baseline smoking characteristics (*p* > 0.05) (Table 1). Both groups exhibited high follow-up rates, with 90.5% (*n* = 182) of nicotine patch users and 90.0% (*n* = 191) of controls re-interviewed at 6 months (*p* = 0.866).

**Table 1:** Demographic and smoking characteristics by group

	Recipients and users of free nicotine patches (*n* = 201)^a^	Control (*n* = 201)^a^	*p*-value
Demographic Characteristics			
Age, mean (SD)	49.6 (12.0)	48.7 (11.2)	0.446
Female, % (n)	50.7 (102)	50.7 (102)	1.000
Married/Common-law, % (n)	53.2 (107)	59.7 (120)	0.191
Employed full- or part- time, % (n)	58.7 (118)	62.2 (125)	0.475
Education Level, % (n)			0.440
Less than high school diploma	24.0 (48)	21.9 (44)	
High school diploma	38.0 (76)	44.3 (89)	
Post-secondary	38.0 (76)	33.8 (68)	
Household Income, % (n)			0.940
< $60,000	65.4 (125)	65.1 (123)	
≥ $60,000	34.6 (66)	34.9 (66)	
Health satisfaction, mean (SD)^b^	3.2 (1.2)	3.2 (1.1)	0.764
Smoking Characteristics			
Cigarettes/day, mean (SD)	18.0 (7.2)	17.9 (6.8)	0.820
FTND score, mean (SD)	5.0 (1.7)	5.0 (1.8)	0.822
Level of Nicotine Dependence, % (n)			1.000
Low	8.0 (16)	8.0 (16)	
Low to Moderate	29.4 (59)	29.4 (59)	
Moderate	56.2 (113)	56.2 (113)	
High	6.5 (13)	6.5 (13)	
Age at first smoking, mean (SD)	14.4 (4.2)	14.8 (3.8)	0.307
Years as smoker, mean (SD)	25.9 (13.3)	24.2 (13.0)	0.199
Number of previous quit attempts, % (n)			0.142
0	5.0 (10)	3.0 (6)	
1–5	63.2 (127)	72.1 (145)	
6 +	31.8 (64)	24.9 (50)	
Past quit methods or aids used, % (n)			
Nicotine replacement therapy (patch/gum/inhaler)	68.6 (131)	64.1 (125)	0.351
Bupropion	34.0 (65)	31.3 (61)	0.565
Varenicline	33.0 (63)	24.6 (48)	0.069
Counselling (individual or group)	7.3 (14)	5.1 (10)	0.370
Acupuncture/hypnosis/herbal remedies	16.2 (31)	16.4 (32)	0.962
Self-help materials	17.3 (33)	17.9 (35)	0.863
Stage of Change			1.000
Precontemplation, % (n)	20.4 (41)	20.4 (41)	
Contemplation, % (n)	39.3 (73)	39.3 (73)	
Preparation, % (n)	40.3 (81)	40.3 (81)	
Confidence in ability to quit, mean (SD)	5.8 (2.6)	5.6 (2.4)	0.428
Importance of quitting now, mean (SD)	7.6 (2.2)	7.7 (2.4)	0.768
Comfort in discussing smoking cessation with family doctor, mean (SD)^c^	8.5 (2.5)	8.7 (2.3)	0.623

Note: SD = standard deviation; FTND = Fagerström Test for Nicotine Dependence

Age, Gender, FTND, and Stage of Change were used to case match control participants to recipients and users of nicotine patches in the experimental group

^a^Sample sizes vary due to missing data on some variables

^b^Health satisfaction was assessed by way of the World Health Organization Quality of Life Instrument (WHOQOL-BREF) [42] question on health satisfaction over the past 2 weeks, where participants were asked to rate how satisfied they are with their health on a 5-point Likert scale of 1 (very dissatisfied) to 5 (very satisfied)

^c^Comfort in discussing smoking cessation with family doctor was assessed on a Likert scale of 1 (not at all comfortable) to 10 (very comfortable). This question was asked only of those in the preparation and contemplation stages of change

At baseline, individuals in the preparation and contemplation stages of change (*n* = 320) were asked whether they thought their doctor was aware of their interest in quitting smoking. Of this subgroup, 88.1% (*n* = 141) of eventual nicotine patch recipients and 90.0% (*n* = 144) of those in the control group had a family physician. Across both groups, three quarters (75.1%) had endorsed that their family doctor was aware, and of those, an average of 87.7% reported that their doctor encouraged them to quit. No differences in rates were noted between groups.

### Physician interaction

Among those with a family physician at the 8 week follow-up (*n* = 177 per group; 88% of entire sample), 26.0% of nicotine patch users had informed their physician they had started using their nicotine patches, while 25.4% of control participants had talked to their doctor about smoking (*p* = 1.00). At the 6 month follow-up, among those contacted with a family physician (*n* = 155 per group; 77% of entire sample), nicotine patch users reported greater frequency of discussing smoking with their physician (43.9%), as compared to the control group (30.3%) (*p* = 0.011).

Of nicotine patch users at 8 weeks, 82.1% (*n* = 165) reported to have used some but not all of the nicotine patches provided and 17.9% (*n* = 36) reported to have used all the patches. No differences in physician interaction were observed between those who used all or just some of the provided nicotine patches at both 8 weeks (χ^2^ = 0.96, *p* = 0.327) and 6 month follow-ups (χ^2^ = 0.91, *p* = 0.341).

### Nicotine patch users’ reasons for and against informing physicians of patch initiation

Among individuals in the experimental group, reasons for informing a physician of nicotine patch use were evaluated at the 8 week follow-up (*n* = 48). While a large majority (62.5%) informed their physician of nicotine patch use as part of a visit for an unrelated issue, 39.6% had sought additional support, 18.8% felt obligated, 12.5% had concerns about possible interactions between the patches and other medication or health conditions, 8.3% were advised by a friend or family member, and 8.3% for other reasons. Among nicotine patch users who had not informed their physician at 8 weeks of their nicotine patch use (*n* = 133) on the other hand, the top rated reason for not informing a physician of nicotine patch use was that they did not think they should have or needed to (56.2%). Other reasons were that it is time consuming to visit a doctor (52.6%), smoking is not a serious medical problem (27.8%), felt they could quit without physician help (26.3%), did not want to use prescription drugs (24.8%), have not seen a doctor (13.5%), believed that doctors can’t help quit smoking (11.3%), and other (15.8%). Separately, reasons for and against informing a family physician of nicotine patch use were not mutually exclusive.

### Physician assistance offered

Of participants who used the freely provided nicotine patches and informed their physician of doing so by the 8 week follow-up (*n* = 48), 39.8% were offered additional support by way of a prescription for varenicline or bupropion, 35.4% were given a brief intervention in the form of a smokers helpline number or pamphlet, 25% were provided with or offered counselling, 18.8% were encouraged to use nicotine gum or inhaler, and 31.3% received none of the above.

Of participants in the control group who had talked about smoking with their doctor by the 8 week follow-up (*n* = 48), 83.3% (*n* = 40) were encouraged to quit. Of these, 50% were offered varenicline or bupropion, 42.5% were given a brief intervention in the form of a smokers’ helpline number or pamphlet, 32.5% were encouraged to use NRT (nicotine patch, gum or inhaler), 15% received or were referred to counselling, and 22.9% received none of the above.

**Fig. 1:**
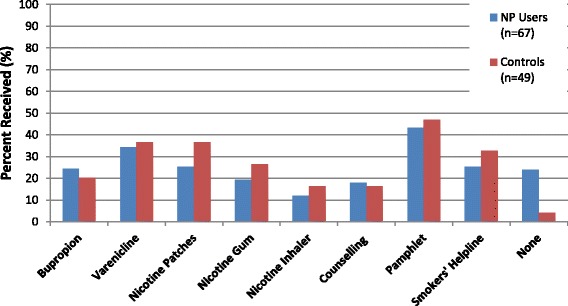
Physician assistance offered between 8 week and 6 month follow-ups. Note: NP, nicotine patch

At the 6 month follow-up, 91.8% (*n* = 67) of nicotine patch users and 92.5% of (*n* = 49) of controls who had talked to their family physician about smoking reported they had been encouraged to quit. Figure 1 depicts proportions of assistance offered by group among those who have talked to their physician about smoking.

### Impact of physician interaction on smoking cessation

Separate univariate ANOVA tests were conducted to evaluate the effect of group, physician interaction and physician interaction by group on quitting smoking at both 8 weeks (7-day point prevalence abstinence) and 6 months (30-day point prevalence abstinence) and making a serious quit attempt (quitting for 1 day or longer), using an intent to treat approach (Table 2). A main effect of Group was evident for abstinence and making a serious quit attempt at both follow-up periods, such that nicotine patch users exhibited greater cessation rates and serious quit attempts compared to case matched controls. A significant interaction effect between group and physician interaction was observed for making a serious quit attempt at 6 month follow-up. Post-hoc analyses revealed that this interaction effect was driven by two observations: a) nicotine patch users who had discussed cessation with a physician had made serious quit attempts at significantly greater rates (72.6%), compared to controls (49.1%) (χ^2^ = 7.28, *p* = 0.007); and b) within the control group, frequency of making serious quit attempts was higher among those that discussed smoking cessation with a physician at 6 month follow-up (49.1%), compared to those who did not speak with a physician (24.4%) (χ^2^ = 10.39, *p* = 0.001).

**Table 2:** Impact of physician interaction on quitting smoking

	Abstinence (7-day pp)		Serious Quit Attempt	
8-week Follow-up	F	*p*-value	F	*p*-value
Main effect of Group	5.69	0.018	160.11	<0.001
Main effect of Physician Interaction	1.93	0.165	1.97	0.161
Interaction between Group and Physician Interaction	2.96	0.086	0.442	0.506
	Abstinence (30-day pp)		Serious Quit Attempt	
6-month Follow-up	F	*p*-value	F	*p*-value
Main effect of Group	3.99	0.047	59.92	<0.001
Main effect of Physician Interaction	0.29	0.594	3.74	0.054
Interaction between Group and Physician Interaction	0.77	0.382	4.14	0.003

Evaluating quitters at 6 months (using 30 day abstinence point prevalence), no differences were observed between the two groups in physician interaction throughout the study, such that 45% (*n* = 9) of abstainers in the nicotine patch use group had talked to their doctor at any point throughout the study, compared to 62.5% (*n* = 5) of those in the control group (Fisher’s Exact Test, *p* = 0.678).

## Discussion

To our knowledge, this is the only intervention study to date to evaluate the impact of free (to the end user) NRT provision on further help seeking and its associated smoking cessation implications. The study identified that while smokers are generally comfortable in turning to their primary care physicians for smoking-related support, most participants, regardless of whether they received and used nicotine patches, did not discuss cessation with their physician by either the 8 week or 6 month follow-ups. Among nicotine patch users at the 8 week follow-up, predominant reasons for not seeking support were beliefs that they did not need to visit a physician, it is time consuming to do so, and the perception that smoking is not a serious medical problem. Such reluctance to proactive support seeking suggests that most smokers generally hold passive views towards primary care physicians’ role in smoking cessation when considering or initiating quitting. Other research has also documented that while some reluctance to consult a practitioner stems from individuals not seeing smoking as an illness [30, 31], others do not seek physician support due to the perception that they have little to offer or the belief that smoking is not a condition that requires medical help [32].

Of those that did visit their physician however, an overwhelming majority reported that their doctors were active in encouraging them to quit. Again, regardless of whether smokers had received and used free nicotine patches, between 83% and 93% of those that discussed smoking cessation with their doctor throughout the 6 month duration of the study were advised to quit, and over 70% were offered additional or alternative support. While the provision and use of nicotine patches, and possibly just surveying about smoking habits, may aid in stimulating patient-initiated discussion of smoking cessation with primary care physicians, these findings point to an encouraging trend of greater physician compliance with the *advise* and *assist* components of the 5A model, as seen in recent representative U.S. population surveys [33, 34]. They may also be indicative of a shift in practice guideline adherence among specifically Canadian physicians, which were reported in 2012 to offer advice to quit to merely 56% of smokers who had visited them in the past year, and fewer than 30% of surveyed smokers received information about assistance [35]. As recipients and users of nicotine patches in the current study were more likely to discuss smoking with their physicians compared to the no intervention control group, the findings suggest that the provision of free NRT particularly to those who are likely to use it may facilitate opportunities for benefits beyond the direct pharmacological effects of the medication, such as the receipt of physician-assisted brief or supplementary intervention. Indeed, nicotine patch users who had discussed cessation with a physician between the 8 week and 6 month follow-ups were more likely to make a serious quit attempt compared to controls. From a public health perspective, the mailed out provision of free nicotine patches may therefore be effective in not only promoting cessation but also in stimulating conversations and support from health professionals that help towards achieving that goal.

It is important to note that approximately 70% of participants who had used the freely provided nicotine patches by 8 weeks and informed their physicians of doing so, were provided with supplementary support, the most common being prescribed varenicline or bupropion. The majority (73.7%) of nicotine patch users had used only some of the provided NRT, therefore it is most likely that the medications were prescribed subsequent to their discontinuation of nicotine patches. The remaining 26.3% however, reported to have used all their nicotine patches by 8 weeks, in which case it is plausible to suspect that these participants were prescribed the medications while they were still using the nicotine patches, contrary to the recommendations of the National Institute for Health and Care Excellence (NICE) [36] and US Public Health Clinical Practice Guidelines for Treating Tobacco Use and Dependence (for varenicline only) [37]. Nearly one half (45.5%) of those who used all their nicotine patches and informed their physician of doing so were prescribed varenicline or bupropion. The combination of bupropion and NRT has been reported to provide similar benefit as either therapy alone [38], and only until a recent systematic review and meta-analysis [39], combination therapy of varenicline and NRT produced favorable, albeit non-significant effects on cessation [40, 41]. However, in the event that the prescribed cessation medications were utilized, it is unclear what role, if any, that would have had on the observed effects in this study. Further research is necessary to evaluate the effectiveness of combination therapy on cessation outcomes in primary care settings, as well as directly contrast the effectiveness of nicotine patches and combination nicotine patch and varenicline in an open label design.

Several limitations of the current study should be noted. First, as nicotine patch users in the experimental group were asked specifically whether they had informed their physician of patch use at 8 weeks, as opposed to whether they had discussed smoking cessation with their physician, direct comparisons between this group of smokers and control participants could not be made. Second, as we were intent on examining how the receipt and specifically use of free nicotine patches is associated with physician interaction, thus necessitating case control matching, the employed random assignment to condition was compromised and placed limits on our ability to infer causality of intervention. Third, the temporal order of nicotine patch use and physician interaction was not captured, therefore nicotine patch use could have either preceded and caused participants to visit their physician (whether due to side effects or additional support), or some participants may have sought physician advice prior to initiating patch use. It is therefore important to emphasize again that the present findings pertain specifically to an association between use of freely provided nicotine patches and physician support, and are not causal in any regard. Third, recipients of the 5 week course of nicotine patches were advised to talk to their physician or pharmacist if they had additional questions to the included instructions on how to use the patches, in compliance with common pharmacotherapy distribution practices and our research ethics board recommendations. Some individuals however, may have perceived this as direct instructions to seek out physician support. Fourth, asking whether participants had visited a physician and discussed smoking cessation could have been subject to recall bias. Finally, biochemical verification of smoking status could not be effectively executed [23], therefore cessation outcomes are based on self-report data.

## Conclusions

Use of freely distributed nicotine patches promoted further smoking cessation centered discussion with family physicians, compared to non-recipients of NRT. Although this was associated with increased likelihood of making a quit attempt, nicotine patch users as a whole generally did not turn to their physicians for cessation assistance, expressing reluctance in seeking their support. In line with grater adherence to clinical practice guidelines of following the 5A model, promotion of physician capacity in addressing tobacco dependence with patients could be enhanced through non-judgmental, stage-based brief motivational interviewing methods. Future research is warranted to examine whether the free NRT distribution as part of smokers’ helplines also mobilizes additional health-care provider support, in particular among pharmacists, who are generally more easily accessible than physicians.

## Abbreviations

ANOVA: Analysis of variance
FTND: Fagerström test for nicotine dependence
NRT: Nicotine replacement therapy
OTC: Over-the-counter

## References

[1] Davis RM (1988). Uniting physicians against smoking: the need for a coordinated national strategy. JAMA.

[2] Leatherdale ST, Shields M (2009). Smoking cessation: intentions, attempts and techniques. Health Rep.

[3] Stead LF, Bergson G, Lancaster T. Physician advice for smoking cessation. Cochrane Database Syst Rev. 2008:CD000165. 10.1002/14651858.CD000165.pub3 18425860

[4] Aveyard P, Begh R, Parsons A, West R (2012). Brief opportunistic smoking cessation interventions: a systematic review and meta-analysis to compare advice to quit and offer of assistance. Addiction.

[5] Fiore M, Jaen C, Baker T, Bailey W, Benowitz N, Curry S, Dorfman S, Froelicher E (2008). Treating tobacco use and dependence: 2008 update. Clinical practice guidelines. U.S. Department of Health and Human Services.

[6] Cummings SR, Rubin SM, Oster G (1989). The cost-effectiveness of counseling smokers to quit. JAMA.

[7] Anda RF, Remington PL, Sienko DG, Davis RM (1987). Are physicians advising smokers to quit? The patient's perspective. JAMA.

[8] Quinn VP, Stevens VJ, Hollis JF, Rigotti NA, Solberg LI, Gordon N, Ritzwoller D, Smith KS, Hu W, Zapka J (2005). Tobacco-cessation services and patient satisfaction in nine nonprofit HMOs. Am J Prev Med.

[9] Goldstein MG, Niaura R, Willey-Lessne C, DePue J, Eaton C, Rakowski W, Dube C (1997). Physicians counseling smokers. A population-based survey of patients’ perceptions of health care provider-delivered smoking cessation interventions. Arch Intern Med.

[10] Schnoll RA, Rukstalis M, Wileyto EP, Shields AE (2006). Smoking cessation treatment by primary care physicians: an update and call for training. Am J Prev Med.

[11] Goldstein MG, DePue JD, Monroe AD, Lessne CW, Rakowski W, Prokhorov A, Niaura R, Dube CE (1998). A population-based survey of physician smoking cessation counseling practices. Prev Med.

[12] Thorndike AN, Rigotti NA, Stafford RS, Singer DE (1998). National patterns in the treatment of smokers by physicians. JAMA.

[13] Papadakis S, McDonald P, Mullen KA, Reid R, Skulsky K, Pipe A (2010). Strategies to increase the delivery of smoking cessation treatments in primary care settings: a systematic review and meta-analysis. Prev Med.

[14] Twardella D, Brenner H (2007). Effects of practitioner education, practitioner payment and reimbursement of patients’ drug costs on smoking cessation in primary care: a cluster randomised trial. Tob Control.

[15] Salize HJ, Merkel S, Reinhard I, Twardella D, Mann K, Brenner H (2009). Cost-effective primary care-based strategies to improve smoking cessation: more value for money. Arch Intern Med.

[16] Bush TM, McAfee T, Deprey M, Mahoney L, Fellows JL, McClure J, Cushing C (2008). The impact of a free nicotine patch starter kit on quit rates in a state quit line. Nicotine Tob Res.

[17] Miller N, Frieden TR, Liu SY, Matte TD, Mostashari F, Deitcher DR, Cummings KM, Chang C, Bauer U, Bassett MT (2005). Effectiveness of a large-scale distribution programme of free nicotine patches: a prospective evaluation. Lancet.

[18] Tinkelman D, Wilson SM, Willett J, Sweeney CT (2007). Offering free NRT through a tobacco quitline: impact on utilisation and quit rates. Tob Control.

[19] Cummings KM, Fix B, Celestino P, Carlin-Menter S, O'Connor R, Hyland A (2006). Reach, efficacy, and cost-effectiveness of free nicotine medication giveaway programs. J Public Health Manag Pract.

[20] Swartz SH, Cowan TM, Klayman JE, Welton MT, Leonard BA (2005). Use and effectiveness of tobacco telephone counseling and nicotine therapy in Maine. Am J Prev Med.

[21] Kotz D, Brown J, West R (2014). ‘Real-world’ effectiveness of smoking cessation treatments: a population study. Addiction.

[22] Kotz D, Brown J, West R (2014). Prospective cohort study of the effectiveness of smoking cessation treatments used in the “real world”. Mayo Clin Proc.

[23] Cunningham JA, Kushnir V, Selby P, Tyndale RF, Zawertailo L, Leatherdale ST (2016). Effect of mailing nicotine patches to promote tobacco cessation among adult smokers: a randomized clinical trial. JAMA Intern Med.

[24] Cunningham JA, Leatherdale ST, Selby PL, Tyndale RF, Zawertailo L, Kushnir V (2011). Randomized controlled trial of mailed nicotine replacement therapy to Canadian smokers: study protocol. BMC Public Health.

[25] Heatherton TF, Kozlowski LT, Frecker RC, Fagerstrom KO (1991). The Fagerstrom test for nicotine dependence: a revision of the Fagerstrom tolerance questionnaire. Br J Addict.

[26] Prochaska JO, Velicer WF (1997). The transtheoretical model of health behavior change. Am J Health Promot.

[27] Velicer WF, Prochaska JO, Rossi JS, Snow MG (1992). Assessing outcome in smoking cessation studies. Psychol Bull.

[28] Niven DJ, Berthiaume LR, Fick GH, Laupland KB (2012). Matched case-control studies: a review of reported statistical methodology. Clin Epidemiol.

[29] Breslow NE, Day NE (1980). Statistical methods in cancer research; volume 1 - the analysis of case-control studies.

[30] Fu SS, Burgess D, van Ryn M, Hatsukami DK, Solomon J, Joseph AM (2007). Views on smoking cessation methods in ethnic minority communities: a qualitative investigation. Prev Med.

[31] Levinson AH, Borrayo EA, Espinoza P, Flores ET, Perez-Stable EJ (2006). An exploration of Latino smokers and the use of pharmaceutical aids. Am J Prev Med.

[32] Smith AL, Carter SM, Chapman S, Dunlop SM, Freeman B (2015). Why do smokers try to quit without medication or counselling? A qualitative study with ex-smokers. BMJ Open.

[33] King BA, Dube SR, Babb SD, McAfee TA (2013). Patient-reported recall of smoking cessation interventions from a health professional. Prev Med.

[34] Kruger J, O'Halloran A, Rosenthal AC, Babb SD, Fiore MC (2016). Receipt of evidence-based brief cessation interventions by health professionals and use of cessation assisted treatments among current adult cigarette-only smokers: National Adult Tobacco Survey, 2009–2010. BMC Public Health.

[35] Reid J, Hammond D, Ruynard V, Burkhalter R (2014). Tobacco use in Canada: Patternsand trends, 2014 edition. In.

[36] Smoking Cessation Services. NICE public health quideline 10. [https://www.nice.org.uk/guidance/ph10/resources/stop-smoking-services-1996169822917]. Accessed 16 Nov 2016.

[37] Clinical Practice guideline Treating Tobacco Use and Dependence 2008 Update Panel L, and Staff (2008). A clinical practice guideline for treating tobacco use and dependence: 2008 update. Ame J Prev Med.

[38] Stapleton J, West R, Hajek P, Wheeler J, Vangeli E, Abdi Z, O'Gara C, McRobbie H, Humphrey K, Ali R (2013). Randomized trial of nicotine replacement therapy (NRT), bupropion and NRT plus bupropion for smoking cessation: effectiveness in clinical practice. Addiction.

[39] Chang PH, Chiang CH, Ho WC, Wu PZ, Tsai JS, Guo FR (2015). Combination therapy of varenicline with nicotine replacement therapy is better than varenicline alone: a systematic review and meta-analysis of randomized controlled trials. BMC Public Health.

[40] Hajek P, Smith KM, Dhanji AR, McRobbie H (2013). Is a combination of varenicline and nicotine patch more effective in helping smokers quit than varenicline alone? A randomised controlled trial. BMC Med.

[41] Ramon JM, Morchon S, Baena A, Masuet-Aumatell C (2014). Combining varenicline and nicotine patches: a randomized controlled trial study in smoking cessation. BMC Med.

[42] Skevington SM, Lotfy M, O'Connell KA (2004). The World Health Organization's WHOQOL-BREF quality of life assessment: psychometric properties and results of the international field trial. A report from the WHOQOL group. Qual Life Res.

